# Capital connectivity in integrated reports: Datasets from international companies

**DOI:** 10.1016/j.dib.2025.111713

**Published:** 2025-05-27

**Authors:** Nurfarahin Roslan, Norman Mohd Saleh, Zaini Embong, Aziatul Waznah Ghazali, Kamarulzaman Kamarudin

**Affiliations:** a Faculty of Economics and Management, Universiti Kebangsaan Malaysia, 43600 Bangi, Selangor, Malaysia; b Faculty of Accountancy, Universiti Teknologi MARA, 78000 Alor Gajah, Melaka, Malaysia; c Faculty of Electrical Engineering & Technology, Universiti Malaysia Perlis, 02600 Arau, Perlis, Malaysia

**Keywords:** Integrated thinking, Capital connectivity, Integrated Reporting

## Abstract

This article provides a detailed dataset on the extent of capital connectivity disclosed in firms' integrated reports following the introduction of the Integrated Reporting (IR) Framework in 2013. The panel data covers 168 firms across Asia-Australia, Europe, and the Americas over a five-year period from 2018 to 2022. Annual integrated reports are collected and downloaded from the official IR database. Data extraction uses a robotic software, which systematically codes the quantity and sequence of specific capitals in the reports. The software uses a list of keywords related to the six capitals: Financial, Manufactured, Intellectual, Human, Social & Relationship, and Natural. A self-calculated connectivity score is then generated to assess firms’ capital connectivity. This measure is based on the narrative analysis detailed in the research article “Drivers of the Disclosed Connectivity of the Capitals: Evidence from Integrated Reports.” The dataset is a valuable indicator of how committed IR adopters are to achieving connectivity, reflecting the integrative thinking involved in producing the integrated report.

Specifications Table

**Table:** 

Subject	Accounting and Taxation
Specific subject area	Connectivity of information
Type of data	Table (Excel File) Supporting Materials (Excel file)
Data collection	A total of 840 integrated reports from 168 firms across Asia-Australia, Europe, and America for the years 2018 to 2022 were downloaded from the IR database. An automated content analysis technique using Questor Version 1.2.0 (2024) software is employed to extract information from a firm’s integrated report that will be used to measure the firm’s capital connectivity.
Data source location	Asia-Australia, Europe, and America region Official Integrated Reporting database
Data accessibility	Repository name: Mendeley Data Data identification number: 10.17632/jhf57r7rnn.2 Direct URL to data: https://data.mendeley.com/datasets/jhf57r7rnn/2
Related research article	Grassmann, M., Fuhrmann, S., & Guenther, T. W. (2019). Drivers of the disclosed “connectivity of the capitals”: evidence from integrated reports. Sustainability Accounting, Management and Policy Journal, 10(5), 877–908. 10.1108/SAMPJ-03-2018-0086

## 1 Value of the Data

• The dataset provides preliminary insights into the connectivity of information disclosed by IR adopters, distinguishing it from other studies that typically concentrate on the amount and level of disclosures. The capital connectivity score captures the quantity and sequence interaction between several types of capital. The data spans multiple firms and regions, offering a comprehensive view that can reveal trends and patterns not visible in isolated datasets.
• The dataset serves as a valuable resource for policymakers in crafting regulations and standards related to integrated reporting. By understanding the degree of capital connectivity in firms' reports, policymakers can proactively take necessary action to encourage adopters to achieve full-scale integration instead of just ticking the IR boxes.
• For IR adopters, the capital connectivity data serves as a benchmark, allowing firms to compare against peers and identify areas for improvement. It enables firms to refine their reporting and better showcase the linkages between different types of capital. This information aids in evaluating firms’ long-term sustainability.

## 2 Background

Significant progress has been made in understanding corporate transparency through voluntary reporting practices [1,2]. However, despite the existence of multiple standards for voluntary reporting, current practices are often criticized for being excessively detailed and disjointed [3,4]. Recent evidence points to a problem of uninformative clutter, where excessive, irrelevant, and redundant information can negate the positive effects of disclosure. To address the shortcomings of traditional reporting, the International Integrated Reporting Council (IIRC) introduced Integrated Reporting (IR) as a new approach that combines financial and sustainability reporting into a single, concise, and coherent document.

IR is distinguished from other reporting initiatives by its emphasis on the core concept of capital connectivity, derived from integrative thinking. Unlike traditional reporting focusing primarily on financial capital, IR adopts a broader perspective on ‘inclusive capital,’ integrating financial, manufactured, intellectual, social and relationship, human, and natural capitals. Given the growing importance of IR in contemporary corporate practices, this article provides data that examines the ‘quantity and types’ of capital reported and highlights the ‘linkage’ by focusing on the ‘sequence’ of capital disclosed within the integrated report.

## 3 Data Description

The panel dataset provides information on the degree of capital connectivity disclosed in integrated reports from 80 Asia-Australian firms, 78 European firms, and 10 American firms from 2018 to 2022. The IIRC identified these years as significant for momentum and global adoption, according to the IIRC's strategic phases [5]. The sample includes firms from eleven different sectors, as defined by the Global Industry Classification Standards (GICS). The dataset focuses on international firms that voluntarily adopt integrated reporting, with these reports available in the IR database. As of March 2023, a total of 343 firm’s data across Asia-Australia, European and the American regions are identified. Of these 343 firms, 48 firms are excluded due to the unavailability of the annual integrated report, and 18 firms are removed because their reports were not in English language. As a result, the annual sample available stands at 277 firms [6]. is referred to determine the minimum annual sample size. Accordingly, a population size of 280 requires a minimum threshold of 162. After conducting rigorous report collection and verification, hence, the final sample ready to be analyse was 168 for one year period, obtaining a total firm-year observation of 840. A stratified random sampling technique was employed to ensure that the sample accurately represents the population. For consistency and comparability, the dataset includes the same companies that produce integrated reports throughout 2018 until 2022. Details on the sample selection procedure and data distribution are presented in Table 1, Table 2, Table 3.

**Table 1:** Sample selection procedure.

Particular/ Region	Asia -Australia	Europe	America	Total
Total adopters listed in the IIRC database	153	161	29	**343**
Less: Integrated report not available	(17)	(24)	(7)	**(48)**
Less: Report not in English	(4)	(9)	(5)	**(18)**
Report available in the IIRC database	132	128	17	**277**
**(%) distribution across regions**	**48 %**	**46 %**	**6 %**	
Final sample (based on Krejcie & Morgan (1970)	80	78	10	**168**
Total firm-year observations (2018-2022)	**840**

**Table 2:** Descriptive statistics for sample firms based on the GICS sector.

GICS Sector	Freq.(Companies)	Percent (%)	Cum. (%)
Communication Services	9	5.36	5.36
Consumer Discretionary	13	7.74	13.1
Consumer Staples	16	9.52	22.62
Energy	3	1.79	24.4
Financials	26	15.48	39.88
Health Care	8	4.76	44.64
Industrials	31	18.45	63.1
Information Technology	16	9.52	72.62
Materials	24	14.29	86.9
Real Estate	9	5.36	92.26
Utilities	13	7.74	100
**Total**	**168**	**100%**

**Table 3:** Descriptive statistics for sample firms based on region/

Region	Freq. (Companies)	Percent (%)	Cum. (%)
Asia-Australasia	80	47.62	47.62
Europe	78	46.43	94.05
America	10	5.95	100
**Total**	**168**	**100%**	

## 4 Experimental Design, Materials and Methods

An automated content analysis using Questor Version 1.2.0 (2024) captures the degree of capital connectivity disclosed. This robotic software codes the quantity and sequence of specific types of capitals based on the identification of the ‘keywords’ that occurred in the integrated report. In this regard, the coding units, categories, and keywords are developed based on extensive research from the IIRC website, published IR framework, and prior literature [[3], [7], [8]]. Following [3], paragraph is used as the coding unit in which a paragraph can be coded up to six capitals. Even if a specific capital appears multiple time in a paragraph, it will still be considered as one capital. Table and figures are considered as one paragraph. The list of keywords comprises six (6) capital categories covering accumulated terms related to Financial (11 terms), Manufactured (25 terms), Intellectual (23 terms), Human (16 terms), Social & Relationship (36 terms), and Natural (33 terms). During the data analysis stage, these predefined categories are assigned to text, to quantify the occurrence and sequence of specific features. Please refer to Table 4 for the definition of each capital category. To analyse the textual connectivity which refer to the notion of connecting different parts of a text, hence, the conjunctive reach was applied. This approach emphasizes on the close proximity of two or more coded capitals within a text segment, reflecting their interconnection.

**Table 4:** Types of capital categories in the list of keywords developed.

No	Code	Capital Category	Definition (IR Framework)
1	FC	Financial	The pool of funds available for a firm to produce goods or provide services can be obtained through debt, equity, grant financing, or income generated through operations or investments.
2	MC	Manufactured	Manufactured physical objects that are available for firms to produce goods or provide services.
3	IC	Intellectual	Organizational, knowledge-based intangibles, including intellectual capital & organizational capital
4	HC	Human Capital	People’s competencies, capabilities & experience
5	SR	Social & Relationship	Relationships between companies and stakeholders, communities & networking available
6	NC	Natural Capital	All renewable and non-renewable environmental resources and processes that provide goods or services that support an organization's past, current, or future prosperity are included.

**Fig. 1:**
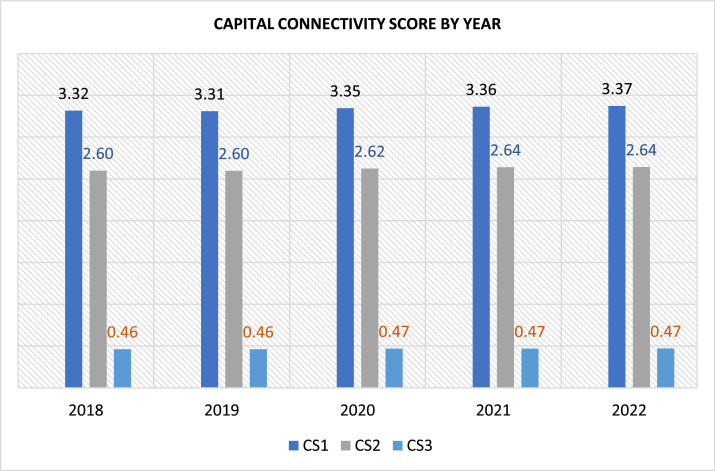
Capital Connectivity Score over the five years (N=840).

**Fig. 2:**
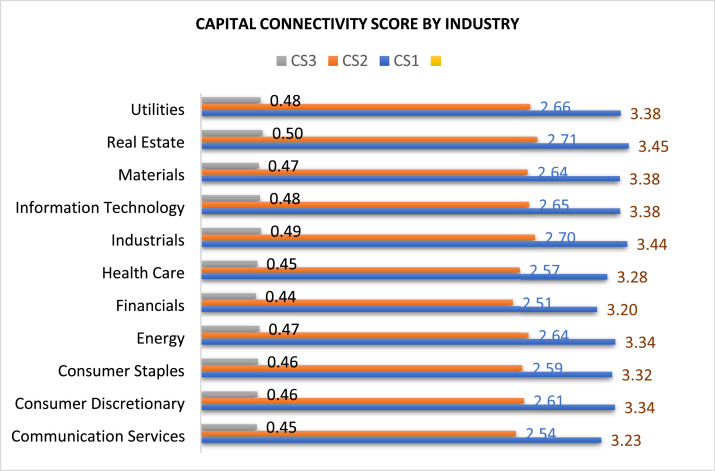
Capital Connectivity Score across Industries.

A self-calculated connectivity score is generated to quantify the capitals connections. This score is derived by counting the average number of different types of capital coded within a specific context in each integrated report. This score ranges from a minimum of one (1) and a maximum of six (6), reflecting the average number of different types of capital codes. Additional scoring is calculated based on the percentage of capital disclosed directly, followed by another capital. The perfect score of 100% indicates that a different capital follows every other capital disclosure, while the lowest possible score of zero means none. Please refer to Table 5 for detailed scoring measurements. Experts from industries and academicians have validated the instrument used to measure capital connectivity. Please refer to supporting material for the instrument validation form. To assess the reliability of the connectivity score, ten (10) integrated reports were randomly selected and manually coded, and the score was calculated based on the manual coding. Weighted Cohen’s Kappa inter-rater reliability test was used to compare Connectivity score (CS) 1 and 2 using manual and automated coding. A coefficient of .99 (CS1) and .94 (CS2) indicates almost perfect agreement between manual and automated coding. In addition, a Pearson correlation is used to compare the manual and automated coding for Connectivity Score 3. A coefficient of .90 (CS3) shows that the manual score is statistically related with automated score at a .01 level of significance. This suggests that the overall score calculated using robotic software is reliable. Fig. 1, Fig. 2 illustrate the mean scores of the IR adopters across various years and industries.

**Table 5:** Measurement for capital connectivity score (CS).

CS	Description	Score
Score 1	The average number of different capitals coded in a surrounding of ± 6 codings (equals on average ±3 paragraphs) around each coding.	**A potential score of min. 1 and max. 6** 1: Average of one capital category coded 2: Average of two diff. capital categories coded 3: Average of three diff. capital categories coded 4: Average of four diff. capital categories coded
Score 2	The average number of different capitals coded in a surrounding of ± 3 codings (equals on average ±2 paragraphs) around each coding.	5: Average of five diff. capital categories coded 6: Average of six diff. capital categories coded
Score 3	The percentage of capital disclosed that is directly followed by a different capital is not the same capital coded twice in a row.	**A potential score of min. 0 and max. 100**

## Limitations

Not applicable

## Ethics Statement

The authors have read and followed the ethical requirements for publication in Data in Brief and confirm that the current work does not involve human subjects, animal experiments, or data collected from social media platforms.

## CRediT Author Statement

**Nurfarahin Roslan:** Conceptualization, Methodology, Data Curation, and Writing -Original Draft. **Norman Mohd Saleh:** Validation, Writing -Review & Editing, Supervision. **Zaini Embong:** Validation and supervision. **Aziatul Waznah Ghazali:** Validation and supervision. **Kamarulzaman Kamarudin:** Software

## Data Availability

Mendeley DataDATASET_IR CONNECTIVITY SCORE (Original data)
